# Association of lunch meat consumption with nutrient intake, diet quality and health risk factors in U.S. children and adults: NHANES 2007–2010

**DOI:** 10.1186/s12937-015-0118-9

**Published:** 2015-12-30

**Authors:** Sanjiv Agarwal, Victor L. Fulgoni, Eric P. Berg

**Affiliations:** NutriScience LLC, 901 Heatherwood Drive, East Norriton, PA 19403 USA; Nutrition Impact LLC, Battle Creek, MI USA; Animal Sciences, North Dakota State University, Fargo, ND USA

**Keywords:** Lunch meat, NHANES, Nutrients, Diet quality, Health risk factors

## Abstract

**Background:**

Consumption of lean meat is recommended as part of healthy diet by *Dietary Guidelines for Americans, 2010*. Lunch meats are precooked or cured meats typically used in sandwiches and are also called as cold cuts or deli meat.

**Objective:**

The purpose of the study was to examine the association of lunch meat consumption with nutrient intake, diet quality, and physiological measures in children (age 2–18 years; *n* = 5,099) and adults (age 19 years and older; *n* = 10,216) using a large, nationally representative database.

**Methods:**

Lunch meat consumers were defined as those consuming any amount of lunch meat during a 24-h recall and association with nutrient intake, diet quality (Healthy Eating Index (HEI)-2010 score) and physiological measures were evaluated using the National Health and Nutrition Examination Survey (NHANES), 2007–2010.

**Results:**

The lunch meat consumers (both children and adults) had higher intakes of calories, protein, calcium, potassium, sodium and saturated fat (for adults only) compared to non-consumers. Lunch meat intake was also associated with higher intake of meat/poultry/fish food group in both children and adult consumers than non-consumers. There was no difference in total HEI-2010 scores comparing lunch meat consumers and non-consumers in children or adults. However, HEI components scores for total fruit, whole fruit (children only), whole grains, dairy and total protein foods were significantly higher, and for greens & beans (adults only), seafood and plant protein, fatty acid ratio and sodium were significantly lower in children and adult lunch meat consumers compared to non-consumers. There were no significant differences in physiological measures or in the odds ratios of health related conditions between lunch meat consumers and non-consumers in children or adults.

**Conclusions:**

The results of this study may provide insight into how to better utilize lunch meats in the diets of U.S. children and adults.

## Background

The *Dietary Guidelines for Americans, 2010* recommend consumption of lean meat as part of an overall healthy diet [1]. MyPlate (ChooseMyPlate.gov) recommends intake from the meat and beans group ranges from 2 to 6.5 ounce (oz.) equivalents depending on age, gender, and physical activity [2]. Meat is an important source of high quality protein and several key micronutrients including iron, zinc, and B-vitamins in American diet [1–8]. The bioavailability of iron and folate from meat is higher than from plant products such as grains and leafy green vegetables [3, 9].

Lunch meats are precooked or cured meats that are sliced for use in sandwich or salad toppings. They are also referred to as “cold cuts” or “deli meat”. There are three types of lunch meats: a) Whole cut – a section of whole muscle that is cooked, flavored/spiced and sliced such as roast beef or corned beef; b) Sectioned/formed –meat trimmings or flakes bound together to form restructured meat products such as multi-part turkey breast or cooked ham; and c) Processed meat – which include fine or coarsely ground meat products such as sausages or emulsified products such as bologna and hot dogs. The recently released Scientific Report of the Dietary Guidelines Advisory Committee 2015 indicated that dietary patterns consisting of lower consumption of red and processed meat were associated with positive health outcomes [10].

The purpose of the present study was to assess the association of lunch meat intake with nutrient intake, diet quality and physiological measures associated with health risk factors in children and adults using a large nationally representative database.

## Methods

### Subjects

National Health and Nutrition Examination Survey (NHANES), a large dietary survey of a nationally representative sample of the non-institutionalized US population, was used to assess lunch meat intake [11]. The NHANES data are collected and released by the National Center for Health Statistics (NCHS) of the Center for Disease Control and Prevention, every two years. All participants or proxies (i.e., parents or guardians) provided written informed consent and the Research Ethics Review Board at the NCHS approved the survey protocol. Dietary intake data with reliable 24-h recall dietary interviews (day 1 data only) using United States Department of Agriculture’s (USDA) automated multiple-pass method were used. The data from NHANES 2007–2008 and 2009–2010 were combined for the analyses [11]. The combined sample included 5,099 children age 2–18 years old and 10,216 adults age 19 years and older excluding pregnant and/or lactating females and those with incomplete or unreliable 24-h recall data.

### Estimation of intake

Lunch meat intakes were assessed using a total of thirty nine USDA food codes for typical lunch meats (Table 1) [12, 13]. These food codes were also used to find food codes containing luncheon meat as an ingredient. Foods such as stews containing small amounts (<7 %) of luncheon meats as ingredient were not considered as lunch meat. Lunch meat consumers were defined as those consuming any amount of lunch meat (from 39 food codes) during a 24-h recall. Energy and nutrient intake were determined using the USDA Nutrient Database for Standard Reference Releases 22 and 24 in conjunction with the Food & Nutrient Database for Dietary Studies versions 4.1 and 5.0, for NHANES 2007–2008 and NHANES 2009–2010 participants respectively [12–15]. The USDA Food Patterns Equivalents Database (FPED) [16] was used to calculate intake MyPlate [17] servings. The FPED translates dietary recall data into equivalent servings of the seven MyPlate major food groups and corresponding subgroups. The number of MyPlate servings was aggregated over all foods consumed during the 24-hour recall to calculate the MyPlate food group intakes per day.

**Table 1:** USDA food codes for lunch meats and their description [12, 13]

Food code	Description	Food code	Description
21420100	Beef, sandwich steak (flaked, formed, thinly sliced)	25230310	Chicken or turkey loaf, prepackaged or deli, luncheon meat
21603000	Beef, pastrami (beef, smoked, spiced)	25230410	Ham loaf, luncheon meat
22311450	Ham, prosciutto	25230430	Ham and cheese loaf
25220010	Cold cut, NFS	25230510	Ham, luncheon meat, chopped, minced, pressed, spiced, not canned
25220390	Bologna, beef, low fat	25230520	Ham, luncheon meat, chopped, minced, pressed, spiced, low fat, not canned
25220400	Bologna, pork and beef	25230530	Ham and pork, luncheon meat, chopped, minced, pressed, spiced, canned
25220410	Bologna, NFS	25230540	Ham, pork and chicken, luncheon meat, chopped, minced, pressed, spiced, canned
25220420	Bologna, Lebanon	25230550	Ham, pork, and chicken, luncheon meat, chopped, minced, pressed, spiced, canned, reduced sodium
25220430	Bologna, beef	25230560	Liverwurst
25220440	Bologna, turkey	25230610	Luncheon loaf (olive, pickle, or pimiento)
25220470	Bologna, beef, lower sodium	25230710	Sandwich loaf, luncheon meat
25220480	Bologna, chicken, beef, and pork	25230790	Turkey ham, sliced, extra lean, prepackaged or deli, luncheon meat
25220500	Bologna, beef and pork, low fat	25230800	Turkey ham
25221500	Salami, NFS	25230810	Veal loaf
25221510	Salami, soft, cooked	25230820	Turkey pastrami
25221520	Salami, dry or hard	25230840	Turkey salami
25230110	Luncheon meat, NFS	25230900	Turkey or chicken breast, prepackaged or deli, luncheon meat
25230210	Ham, sliced, prepackaged or deli, luncheon meat	25230905	Turkey or chicken breast, low salt, prepackaged or deli, luncheon meat
25230220	Ham, sliced, low salt, prepackaged or deli, luncheon meat	25231110	Beef, sliced, prepackaged or deli, luncheon meat
25230230	Ham, sliced, extra lean, prepackaged or deli, luncheon meat		

### Estimation of diet quality

Diet quality was calculated using the Healthy Eating Index (HEI) -2010 which has 12 components, each representing a different aspect of diet quality [18]. HEI - 2010 scores were estimated using day 1 dietary intake data. The SAS code used to calculate HEI - 2010 scores was downloaded from the USDA website [19].

### Estimation of physiological markers of risk

Health indices evaluated included body weight, body mass index (BMI) and Z score for children [20], as well as BMI, waist circumference, blood pressure, fasting plasma glucose, fasting plasma insulin, c–reactive protein, fasting triglycerides, total cholesterol, LDL-cholesterol (fasting), HDL-cholesterol, and apolipoprotein B, and risk of metabolic syndrome for adults using NHANES standard protocols [11]. For a variety of reasons, not all individuals have values for all tests (see tables for sample numbers). Metabolic syndrome was defined using the NHLBI Adult Treatment Panel III criteria [21], namely having three or more of the following risk factors: abdominal obesity (waist circumference >102 cm for males and >88 cm for females); hypertension (BP systolic ≥130 mmHg or BP diastolic ≥85 mmHg or taking anti-hypertensive medications); low HDL-cholesterol (<40 mg/dL for males and <50 mg/dL for females); high triglycerides (≥150 mg/dL or taking anti-hyperlipidemic medications); high fasting glucose (≥110 mg/dL or taking insulin or other hypoglycemic agents).

### Statistical analysis

All analyses were performed using SAS 9.2 (SAS Institute, Cary, NC) and SUDAAN 11 (RTI, Research Triangle Park, NC) to adjust the variances for the complex sample design of NHANES and thus survey weights, strata and primary sampling units were used in all calculations. Day one dietary weights were used in all intake analyses, while the Mobile Examination Center weights were used for physiological variables except where the outcome was a fasting lab variable in which case fasting subsample weights were used. Least square means (Mean), standard errors of the mean (SEM), via regression analyses were determined for energy and nutrient intakes, food group intake, diet quality, and physiological markers of metabolic disease risk in lunch meat consumers and non-consumers. Food group/nutrient intakes were adjusted for age (even within each age group), gender, ethnicity, poverty income ratio, self-reported physical activity level, smoking status, alcohol intake (only for adults), and energy intake (except for energy intake). Diet quality was adjusted for the same covariates but without energy intake as HEI scores are already adjusted for energy intake. Physiological variables were adjusted for age, gender, ethnicity, poverty-income ratio, self-reported physical activity level, smoking status, alcohol intake (only for adults) and BMI (for non-weight related variables).

## Results

Approximately 19.4 % adults (age 19 years and older) and 17.8 % children (age 2–18 years) were lunch meat consumers. In both children and adults, there were no major demographic differences between lunch meat consumers and non-consumers except that there were 12 % fewer adult female consumers (46.2 % female adult consumers versus 52.6 % female non-consumers, *P* < 0.0001).

There were significant differences in nutrient intakes between the lunch meat consumers and the non-consumers (Table 2). Compared with non-consumers in children, lunch meat consumers had significantly higher (*P* < 0.01) intakes of calories (7.3 %), and energy adjusted daily intakes of protein (8.3 %), calcium (13 %), potassium (5.5 %), thiamin (7.3 %) and sodium (15.6 %). Similarly adult lunch meat consumers also had significantly higher (*P* < 0.01) intakes of calories (4.4 %), and energy adjusted intakes of protein (6.3 %), saturated fatty acids (3.6 %), calcium (14.3 %), potassium (6.2 %), thiamin (11.1 %), and sodium (18.3 %) compared to adult non-consumers. Adult lunch meat consumers also had significantly lower (*P* < 0.01) intakes of monounsaturated fatty acids (MUFA) (−3.6 %) and polyunsaturated fatty acids (PUFA) (−4.5 %) compared to non-consumers. The intake of other nutrients was not significantly different among lunch meat consumers and non-consumers (Table 2).

**Table 2:** Energy and nutrient intakes in children (*n* = 5099) and adult (*n* = 10216) lunch meat consumers and non-consumers (NHANES 2007–2010, gender combined data). Values are means ± SEM. Values are adjusted for age, gender, ethnicity, poverty income ratio, physical activity level, current smoking status, alcohol (only for adults), and kcal (except for energy)

Variables	Children (2–18 years old)			Adults (19 years and older)		
	Non-consumers	Consumers	*P* Value for difference	Non-consumers	Consumers	*P* Value for difference
Energy (kcal)	1873 ± 17	2009 ± 42	0.0022	2115 ± 14	2209 ± 27	0.0029
Protein (gm)	66.6 ± 0.4	72.1 ± 1.0	<0.0001	81.6 ± 0.4	86.8 ± 0.8	<0.0001
Carbohydrate (gm)	255 ± 1	250 ± 2	0.0236	258 ± 1	254 ± 2	0.0122
Dietary fiber (gm)	13.0 ± 0.2	12.9 ± 0.2	0.7796	16.5 ± 0.3	15.7 ± 0.3	0.0147
Total sugars (gm)	127 ± 1.3	122 ± 2	0.0201	118 ± 1	114 ± 2	0.0253
Total fat (gm)	69.6 ± 0.4	69.6 ± 1.0	0.9627	80.3 ± 0.4	79.6 ± 0.6	0.2920
MUFA (gm)	25.1 ± 0.2	24.2 ± 0.5	0.0647	29.4 ± 0.2	28.4 ± 0.3	0.0016
PUFA (gm)	14.0 ± 0.2	13.8 ± 0.3	0.4677	17.5 ± 0.1	16.8 ± 0.2	0.0008
SFA (gm)	24.4 ± 0.2	25.3 ± 0.6	0.1387	26.3	27.2 ± 0.3	0.0021
Cholesterol (mg)	210 ± 3	228 ± 8	0.0269	287 ± 4	277 ± 5	0.0944
Calcium (mg)	1006 ± 12	1136 ± 29	0.0006	953 ± 9	1090 ± 16	<0.0001
Iron (mg)	13.5 ± 0.2	13.8 ± 0.4	0.3901	15.2 ± 0.1	15.3 ± 0.2	0.5789
Magnesium (mg)	228 ± 2	235 ± 5	0.1177	299 ± 3	302 ± 3	0.4044
Potassium (mg)	2141 ± 22	2258 ± 37	0.0020	2674 ± 22	2839 ± 26	<0.0001
Sodium (mg)	2966 ± 28	3427 ± 52	<0.0001	3494 ± 18	4132 ± 43	<0.0001
Vitamin A (μg)	592 ± 10	591 ± 19	0.9744	621 ± 9	637 ± 15	0.2720
ß-carotene (μg)	1171 ± 61	1136 ± 137	0.8192	2081 ± 62	2002 ± 116	0.4936
Thiamin (mg)	1.5 ± 0.02	1.6 ± 0.03	0.0012	1.6 ± 0.02	1.8 ± 0.03	<0.0001
Total Folate (μg)	360 ± 5	361 ± 17	0.9696	409 ± 5	403 ± 6	0.4370
Vitamin B6 (mg)	1.7 ± 0.03	1.8 ± 0.1	0.2082	2.0 ± 0.02	2.1 ± 0.04	0.0302
Vitamin C (mg)	78.6 ± 1.9	86.5 ± 4.4	0.0763	86.2 ± 2.1	83.5 ± 2.3	0.2940

*MUFA* monounsaturated fatty acids, *PUFA* polyunsaturated fatty acids, *SFA* saturated fatty acids

Intake of lunch meat was also associated with significant differences (*P* < 0.01) in specific MyPlate food groups (Table 3). Significantly higher intakes of meat/poultry/fish (31.8 %) and whole grain (37.1 %), and lower intake of added sugars (−7.5 %) were observed among children consuming lunch meats compared to non-consumers. Among adults, consumers of lunch meat had higher intakes of meat/poultry/fish (30.6 %), whole grain (30.7 %), dairy (17.9 %) and grains (5.9 %), and lower intake of vegetables (−7.6 %) compared to adult non-consumers.

**Table 3:** Intake of MyPlate food groups in children (*n* = 5099) and adult (*n* = 10216) lunch meat consumers and non-consumers (NHANES 2007–2010, gender combined data). Values are means ± SEM. Values are adjusted for age, gender, ethnicity, poverty income ratio, physical activity level, current smoking status, alcohol (only for adults), and kcal

Variables	Children (2–18 years old)			Adults (19 years and older)		
	Non-consumers	Consumers	*P* Value for difference	Non-consumers	Consumers	*P* Value for difference
Total Fruit (cup eq.)	1.08 ± 0.04	1.23 ± 0.06	0.0102	0.99 ± 0.03	1.07 ± 0.04	0.0241
Whole Fruit (cup eq.)	0.67 ± 0.04	0.78 ± 0.06	0.0252	0.67 ± 0.02	0.76 ± 0.03	0.0318
Fruit Juice (cup eq.)	0.41 ± 0.02	0.44 ± 0.04	0.3352	0.31 ± 0.01	0.31 ± 0.03	0.9484
Total Vegetable (cup eq.)	0.90 ± 0.03	0.81 ± 0.03	0.0362	1.58 ± 0.03	1.46 ± 0.03	0.0042
Total Grain (oz eq.)	6.32 ± 0.07	6.67 ± 0.15	0.0286	6.37 ± 0.06	6.75 ± 0.10	0.0027
Whole Grain (oz eq.)	0.53 ± 0.02	0.73 ± 0.04	0.0003	0.72 ± 0.03	0.94 ± 0.04	<0.0001
Total Dairy (cup eq.)	2.17 ± 0.04	2.33 ± 0.10	0.2019	1.61 ± 0.02	1.90 ± 0.04	<0.0001
Milk (cup eq.)	1.44 ± 0.03	1.25 ± 0.05	0.0129	0.85 ± 0.02	0.86 ± 0.03	0.9151
Meat/Poultry/Fish (oz eq.)	3.21 ± 0.08	4.23 ± 0.12	<0.0001	4.70 ± 0.06	6.14 ± 0.08	<0.0001
Added Sugar (tsp eq.)	19.4 ± 0.3	17.9 ± 0.4	0.0092	18.5 ± 0.4	17.5 ± 0.5	0.0264

Despite some differences in food groups intake, there was no difference in dietary quality (measured by the HEI-2010) comparing lunch meat consumers and non-consumers for children (HEI-2010 scores difference between consumers and non-consumers: −0.30, *P* = 0.6187) or adults (HEI-2010 scores difference between consumers and non-consumers: −0.61, *P* = 0.2010) (Table 4). When the data were further analyzed for different age groups for children and adults: young children age 2–9 years, adolescent age 9–19 years; adults 19–50 years and adults 51 years and older; and for males and females separately there were still no significant differences (*P* > 0.01) in HEI 2010 scores between consumers and non-consumers (Table 4).

**Table 4:** Healthy Eating Index (HEI) – 2010 total score for children and adult lunch meat consumers and non-consumers (NHANES 2007–2010) by age and gender subgroups. Values are means ± SEM. Values are adjusted for age, gender, ethnicity, poverty income ratio, physical activity level, current smoking status, alcohol (only for adults), and kcal

Population subgroup	N	Total HEI 2010 score		
		Non-consumer	Consumer	*P* Value
Children age 2–18 years				
Gender combined	5099	46.02 ± 0.41	45.71 ± 0.56	0.6187
Female	2455	46.14 ± 0.54	46.44 ± 0.74	0.7489
Male	2644	45.88 ± 0.49	45.06 ± 0.83	0.3664
Young Children age 2–8 years				
Gender combined	2476	50.00 ± 0.43	48.53 ± 0.71	0.0782
Female	1163	49.96 ± 0.64	49.39 ± 0.92	0.6418
Male	1313	50.04 ± 0.57	47.32 ± 1.24	0.0776
Adolescent age 9–18 years				
Gender combined	2623	42.92 ± 0.52	43.75 ± 0.85	0.3518
Female	1292	43.44 ± 0.70	44.55 ± 1.20	0.4152
Male	1331	42.36 ± 0.63	42.99 ± 1.00	0.5198
Adults age 19 years & older				
Gender combined)	10216	49.40 ± 0.43	48.79 ± 0.51	0.2010
Female	5116	50.60 ± 0.56	50.54 ± 0.73	0.9387
Male	5100	48.12 ± 0.38	47.00 ± 0.54	0.0193
Adults age 19–50 years				
Gender combined	5359	46.79 ± 0.48	47.19 ± 0.70	0.5271
Female	2703	47.64 ± 0.63	48.85 ± 1.00	0.2401
Male	2656	45.95 ± 0.48	45.65 ± 0.82	0.6456
Adults age 51 years & older				
Gender combined	4857	53.24 ± 0.53	51.23 ± 0.68	0.0124
Female	2413	54.64 ± 0.58	52.95 ± 0.82	0.1083
Male	2444	51.61 ± 0.54	49.30 ± 0.95	0.0319

Although the total HEI 2010 was similar for lunch meat consumers and non-consumers among children and adults, there were differences in the scores for components of HEI 2010 (Table 5). In children age 2–19 years, HEI components scores were significantly higher (*P* < 0.01) for total fruit (12.6 %), whole fruit (17.4 %), whole grains (30.6 %), dairy (9.0 %), and total protein foods (18.8 %) in lunch meat consumers while component scores for seafood and plant protein (−25.6 %), fatty acid ratio (−18.5 %) and sodium (−38.3 %) were significantly lower (*P* < 0.01) compared to non-consumers. Similarly, among adults, the HEI components scores were significantly higher (*P* < 0.01) for total fruit (9.1 %), whole grains (31.1 %), dairy (16.2 %), and total protein foods (9.8 %) in lunch meat consumers while component scores for greens and beans (−19.0 %), seafood and plant protein (−18.8 %), fatty acid ratio (−12.8 %) and sodium (−41.8 %) were significantly lower (*P* < 0.01) compared to non-consumers (Table 5).

**Table 5:** Healthy Eating Index (HEI) – 2010 total score and component scores of children (*n* = 5099) and adult (*n* = 10216) lunch meat consumers and non-consumers (NHANES 2007–2010 gender combined data). Values are means ± SEM. Values are adjusted for age, gender, ethnicity, poverty income ratio, physical activity level, current smoking status, alcohol (only for adults), and kcal

Variables	Children (2–18 years old)			Adults (19 years and older)		
	Non-consumers	Consumers	*P* Value for difference	Non-consumers	Consumers	*P* Value for difference
HEI-2010 Total Score	46.02 ± 0.41	45.71 ± 0.56	0.6187	49.40 ± 0.43	48.79 ± 0.51	0.2010
Component 1 (Total Vegetables)	2.10 ± 0.04	1.95 ± 0.08	0.0821	3.05 ± 0.04	2.89 ± 0.05	0.0100
Component 2 (Greens & Beans)	0.68 ± 0.05	0.52 ± 0.08	0.0902	1.28 ± 0.05	1.03 ± 0.06	0.0001
Component 3 (Total Fruit)	2.51 ± 0.06	2.83 ± 0.11	0.0059	2.13 ± 0.04	2.32 ± 0.06	0.0039
Component 4 (Whole Fruit)	2.24 ± 0.07	2.63 ± 0.15	0.0095	2.07 ± 0.05	2.28 ± 0.08	0.0212
Component 5 (Whole Grains)	1.97 ± 0.07	2.57 ± 0.13	0.0007	2.23 ± 0.08	2.93 ± 0.10	<0.0001
Component 6 (Dairy)	6.97 ± 0.09	7.60 ± 0.15	0.0013	5.10 ± 0.07	5.92 ± 0.11	<0.0001
Component 7 (Total Protein Foods)	3.46 ± 0.04	4.11 ± 0.05	<0.0001	4.16 ± 0.02	4.57 ± 0.02	<0.0001
Component 8 (Seafood & Plant Protein)	1.37 ± 0.04	1.02 ± 0.09	0.0013	2.07 ± 0.04	1.68 ± 0.07	<0.0001
Component 9 (Fatty Acid Ratio)	3.88 ± 0.07	3.16 ± 0.16	0.0004	4.98 ± 0.06	4.34 ± 0.13	0.0002
Component 10 (Sodium)	5.17 ± 0.11	3.19 ± 0.17	<0.0001	4.39 ± 0.06	2.55 ± 0.09	<0.0001
Component 11 (Refined Grains)	5.30 ± 0.09	5.00 ± 0.20	0.1818	6.21 ± 0.06	6.05 ± 0.15	0.2908
Component 12 (SoFAAS Calories)	10.36 ± 0.13	11.14 ± 0.31	0.0224	11.73 ± 0.17	12.22 ± 0.19	0.0167

There were no differences in any studied physiological measures (body weight, waist circumference, body mass index, systolic blood pressure, diastolic blood pressure, fasting plasma glucose, fasting plasma insulin, C-reactive protein, fasting triglycerides, total cholesterol, LDL- cholesterol, HDL-cholesterol, and apolipoprotein B) associated with lunch meat consumption in children age 2–18 years and in adults age 19 years and older (Table 6). The differences between consumers and non-consumers remained non-significant (*P* > 0.01) when the data was further analyzed for different age groups for children and adults: young children age 2–9 years, adolescent age 9–19 years; adults 19–50 years and adults 51 years and older; and for males and females (data not presented).

**Table 6:** Association of lunch meat consumption with physiological measures in children and adults - NHANES 2007–2010. Values are means ± SEM. Values are adjusted for age, gender, ethnicity, poverty income ratio, physical activity level, current smoking status, alcohol (only for adults) and weight (only for variable not related to weight)

Physiological variables	N	Non-consumer	Consumer	*P* Value
Children age 2–18 years				
BMI Z Score	5046	0.44 ± 0.03	0.48 ± 0.05	0.4464
Adults age 19 years and older				
Weight (kg)	10,108	82.19 ± 0.35	82.08 ± 0.65	0.8859
Body Mass Index (kg/m^2^)	10,097	28.69 ± 0.12	28.73 ± 0.21	0.8631
Waist Circumference (cm)	9,821	98.10 ± 0.30	97.98 ± 0.53	0.8631
BP Diastolic (mm Hg)	9,761	70.63 ± 0.37	70.42 ± 0.44	0.5349
BP Systolic (mm Hg)	9,802	121.28 ± 0.29	121.51 ± 0.43	0.6721
Total cholesterol (mg/dL)	9,604	196.95 ± 0.73	196.35 ± 1.44	0.6160
LDL-cholesterol (mg/dL)	4,280	116.68 ± 0.78	113.83 ± 1.41	0.0539
HDL-cholesterol (mg/dL)	9,604	52.52 ± 0.33	52.57 ± 0.42	0.8991
Triglyceride (mg/dL)	4,359	129.81 ± 1.91	131.96 ± 3.87	0.6182
Apolipoprotein (B) (mg/dL)	4,357	92.34 ± 0.66	90.47 ± 1.03	0.0620
C-reactive protein (mg/dL)	9,636	0.37 ± 0.01	0.42 ± 0.04	0.2105
Glucose, plasma (mg/dL)	4,391	104.59 ± 0.61	106.42 ± 1.29	0.2162
Insulin (μU/mL)	4,332	12.70 ± 0.23	12.28 ± 0.27	0.2582

The odds ratios of health related conditions were also similar (overlapping 95 % CI) for lunch meat consumers compared to non-consumers for both children and adults (Table 7). Lunch meat consumers did not show any significant differences in odds ratios compared with non-consumers even when the data was further analyzed for different age groups for children: young children age 2–9 years, adolescent age 9–19 years; for adults: age 19–50 years and 51 years and older; and for males and females (data not presented).

**Table 7:** Association of lunch meat consumption with odds ratios of weight/waist status and other risk factors in children and adults - NHANES 2007–2010. Values are adjusted for age, gender, ethnicity, poverty income ratio, physical activity level, current smoking status, alcohol (for adults only), and weight (only for variable not related to weight)

Variables	N	Odds ratio (95 % CI)		
		Non-consumer	Consumer	*P* Value
Children age 2–18 years				
Obese	5046	1.00	1.14 (0.90, 1.44)	0.2577
Overweight	5046	1.00	1.01 (0.70, 1.47)	0.9560
Overweight or Obese	5046	1.00	1.09 (0.85, 1.40)	0.4693
Adults age 19 years and older				
Obese	10,097	1.00	0.97 (0.82, 1.16)	0.7518
Overweight	10,097	1.00	0.89 (0.75, 1.04)	0.1391
Overweight or Obese	10,097	1.00	0.86 (0.74, 1.01)	0.0582
Waist Circumference Elevated	9,821	1.00	0.88 (0.73, 1.05)	0.1519
BP Elevated	9,971	1.00	0.96 (0.78, 1.18)	0.6777
HDL Reduced	9,808	1.00	1.01 (0.88, 1.15)	0.9306
LDL Elevated	4,359	1.00	0.88 (0.70, 1.10)	0.2594
Triglycerides Elevated	4,420	1.00	1.03 (0.83, 1.28)	0.7786
Glucose Elevated	4,443	1.00	1.13 (0.91, 1.40)	0.2699
Metabolic Syndrome	7,229	1.00	0.98 (0.75, 1.28)	0.8699

## Discussion

This is the first report to investigate lunch meat consumption in the U.S. population and explore its relationships with nutrient intake, diet quality and physiological markers of health. In the present study, we combined NHANES 2007–2008 and NHANES 2009–2010 data and the combined data set provided a sample size of over 15 thousand adults and children. Lunch meats, also known as deli meat or cold cuts, are precooked or cured meat that are sliced and used to make a convenient sandwich filling or salad topping. The NHANES 2007–2010 data showed that almost one-fifth of the population (18 % children age 2–18 and 19 % adults age 19 years and older) consumed lunch meat on the day of the recall.

Both adults and children consumers of lunch meat consumed significantly more calories as well as protein compared to their respective non-consumers. Additionally, they consumed more energy adjusted calcium, potassium and thiamine compared to non-consumers. Calcium and potassium are termed as “nutrients of concern” by the *Dietary Guidelines for Americans, 2010* [1]. Current intake of calcium is estimated to be below the Estimated Average Requirement for over 40 % of the population and only about 3 % population is currently consuming more than the Adequate Intake for potassium [10]. Adequate calcium status is important for optimal bone health and potassium helps lower the blood pressure. *Dietary Guidelines for Americans, 2010* has recommended increasing intake of calcium and potassium [1]. Meat (especially lean meat) is considered as one of the most nutrient dense food [6]. Lunch meat consumers also had higher intakes of sodium compared to non-consumers. Excessive sodium intake has been related to high prevalence of high blood pressure [1]. Sodium intake estimated in this study was higher than the recommended 2,300 mg for both adult and children, irrespective of their being consumers or non-consumers. Potassium lowers blood pressure by blunting the adverse effects of sodium on blood pressure. However, it should be noted there were no significant differences in blood pressure between consumers and non-consumers for both adults and children.

The HEI 2010 scores of lunch meat consumers were not significantly different from those of non-consumers for both adults and children. The HEI is a measure of diet quality that indicates compliance/adherence of the diets to the recommendations of *Dietary Guidelines for Americans, 2010*. HEI is commonly used to evaluate diets including subpopulations [22] and food environments [23], to assess changes in the diet quality over time [24] and the efficacy of dietary interventions, and to validate other nutrition research tools and indexes [25]. It has also been used in recent research to understand relationships between nutrients/foods/dietary patterns and health-related outcomes [26–29]. Lunch meat consumers had similar HEI 2010 scores as non-consumers and the differences in HEI 2010 scores remained non-significant even when the data was further analyzed by age (young children, adolescent, adults and older adults) and gender groups (males and females) indicating that the diet quality of lunch meat consumers were similar to non-consumers for every age/gender group. These results suggest that lunch meats do not necessarily decrease average diet quality of adults and children. HEI 2010 has 12 components (9 for adequacy and 3 for moderation) each of which relate to the key recommendations of the *Dietary Guidelines for Americans, 2010*. Although the total HEI scores were not different for lunch meat consumers compared to non-consumers, there were some differences in the subcomponent scores which may provide insight into incorporating lunch meats into diets to help align with Dietary Guidelines Recommendations. For example, lower sodium lunch meat options may be considered and it appears that the use of cheese and whole grains are more common in lunch meat consumers thus improving HEI subcomponent scores for dairy and whole grains, respectively.

In the current study, lunch meat intake was not associated with any physiological measurements including BMI, blood pressure, blood lipids or blood sugar. The recently released Scientific Report of the Dietary Guidelines Advisory Committee 2015 indicated that dietary patterns consisting of lower consumption of red and processed meat were associated with positive health outcomes [10]. In an abstract the International Agency for Research on Cancer experts indicated that 34,000 to 50,000 cancer deaths per year worldwide may be attributable to diets high in processed meat and red meat respectively while air pollution, alcohol intake and tobacco smoking are responsible for 200,000, 600,000 and 1 million deaths per year worldwide respectively [30, 31]. The expert report concluded that each 50 g portion of processed meat eaten daily may increase the risk of colorectal cancer by 18 %, [30, 31]. It is impossible to accurately discern the contribution of one single food consumed with causation of cancer because it is impossible to separate an individual food from the confounding interactions of other potential carcinogenic “hazards” experienced over the course of a lifetime. Intake of processed meat was found to be associated with a higher risk of coronary heart disease (CHD) and type-2 diabetes [32]. In a meta-analysis, consumption of processed meats was associated with higher incidence of CHD and diabetes mellitus [33]. High intake of processed meat was also implicated with increased risk of early death, in particular due to cardiovascular diseases and cancer, in a recently published European epidemiological study [34]. However, most observational studies reported only a small increase in relative risk [6]. Industry efforts for the past 10 years have focused on simplification of ingredient lists. This “clean labeling” effort has resulted in significant changes regarding processing techniques applied to product development of luncheon meats. In the present study, we did not find any significant differences in physiological measures or in the odds ratio of all studied health related conditions between lunch meat consumers and non-consumers (in fact, lunch meat consumers had better average physiological measures for lipids and overweight/obesity risk in our findings.) Whether the lack of significant effect on physiological parameters is a result of using NHANES, an observational study; effect of presence of other dietary components such as fruits and vegetables, whole grain etc. in the diet of consumers; or whether lunch meats have evolved since the previously mentioned studies were published will have to await further research.

A limitation of this study is that cross-sectional studies cannot be used to determine cause and effect. Additionally, 24-h dietary recalls rely on participants’ memory to self-report dietary intakes; and therefore data are subject to misreporting. Also the data used in this study was based on single 24-h dietary recall. Strengths of this study included the use of large nationally representative sample achieved through combining several sets of NHANES data releases and adjusting for numerous covariates, but even with these covariates some residual confounding may still exist.

Future research might consider comparing lunch meat consumers, with consumers of other meats and/or non-consumers of meat. Additionally, when further data are available it might also be meritorious to conduct analyses comparing particular types of lunch meats (e.g., whole cut versus sectioned/formed, versus processed meat). Other work might examine further other items consumed with lunch meats and whether more healthy items could be identified.

## Conclusion

In conclusion, results from this study suggest that lunch meat intake did not affect the overall diet quality while differences in certain subcomponents scores (dairy, whole grains and sodium) suggest there may be ways to incorporate lunch meats into healthy dietary patterns.

## Abbreviations

BMI: Body Mass Index
HEI: Healthy Eating Index
MUFA: Mono unsaturated fatty acids
NHANES: National Health and Nutrition Examination Survey
PUFA: Poly unsaturated fatty acids
SEM: Standard errors of the mean
SFA: Saturated fatty acids
